# Household clustering of asymptomatic malaria infections in Xepon district, Savannakhet province, Lao PDR

**DOI:** 10.1186/s12936-016-1552-7

**Published:** 2016-10-18

**Authors:** Tiengkham Pongvongsa, Daisuke Nonaka, Moritoshi Iwagami, Masami Nakatsu, Panom Phongmany, Futoshi Nishimoto, Jun Kobayashi, Bouasy Hongvanthon, Paul T. Brey, Kazuhiko Moji, Toshihiro Mita, Shigeyuki Kano

**Affiliations:** 1 Savannakhet Provincial Health Department, Phonsavangnuea Village, Kaysone-Phomvihan District, Savannakhet, Lao PDR; 2 Department of Molecular and Cellular Parasitology, School of Medicine, Juntendo University, 2-1-1 Hongo, Bunkyo-ku, Tokyo, 113-8421 Japan; 3 SATREPS Project for Parasitic Diseases, Vientiane, Lao PDR; 4 Department of Global Health, School of Health Sciences, University of the Ryukyus, 207 Uehara, Nishihara-cho, Okinawa, 903-0215 Japan; 5 Department of Tropical Medicine and Malaria, Research Institute, National Center for Global Health and Medicine, 1-21-1 Toyama, Shinjuku-ku, Tokyo, 162-8655 Japan; 6 Graduate School of Tropical Medicine and Global Health, Nagasaki University, 1-12-4 Sakamoto, Nagasaki-shi, Nagasaki, 852-8523 Japan; 7 Center of Malariology, Parasitology and Entomology, Ministry of Health, Vientiane, Lao PDR; 8 Institut Pasteur du Laos, Sisattanak District, Vientiane, Lao PDR

**Keywords:** Asymptomatic infections, Sub-microscopic infections, Malaria elimination, Active case detection, Laos

## Abstract

**Background:**

In the Lao PDR, malaria morbidity and mortality have remarkably decreased over the past decade. However, asymptomatic infections in rural villages contribute to the on-going local transmission. The primary objective of this study was to explore the characteristics of infections in a malaria-endemic district of the Lao PDR. The specific objectives were to investigate the prevalence and species of malaria parasites using molecular methods and to assess individual and household parasite levels and the characteristics associated with malaria infection.

**Methods:**

The study population included 870 participants from 236 households in 10 villages of the Xepon district. Interviews, blood examinations and body temperature measurements were conducted between August and September 2013. A multilevel logistic regression model, with adjustment for clustering effects, was used to assess the association between predictor variables and an outcome variable (malaria infection status as principally determined by PCR). The predictive factors included individual-level factors (age, gender, past fever episode, and forest activity during night time) and household-level factors (household member size, household bed net usage/density and a household with one other malaria-infected member).

**Results:**

Fifty-two participants (including 26 children) tested positive (positive rate: 6.0 %): *Plasmodium falciparum* mono-infection was the most common infection (n = 41, 78.8 %), followed by *P. falciparum* and *Plasmodium vivax* mixed infections (n = 9, 17.3 %). The majority of infected participants (n = 42, 80.8 %) had no fever episodes in the two previous weeks or a measurable fever (>37 °C) at the time of survey. Living in a household with one other malaria-infected member significantly increased the odds of infection (odds ratio 24.33, 95 % confidence interval 10.15–58.32). Among the 40 households that had at least one infected member, nine households were responsible for 40.4 % of the total infections.

**Conclusions:**

*Plasmodium vivax* was detected more frequently than it was reported from the district hospital. Most infections were asymptomatic and sub-microscopic and were highly clustered within households. To further eliminate malaria in Xepon and other similar settings in the country, the National Malaria Control Programme should consider household-based strategies, including reactive case detection targeting the household members of index cases.

**Electronic supplementary material:**

The online version of this article (doi:10.1186/s12936-016-1552-7) contains supplementary material, which is available to authorized users.

## Background

The Lao People’s Democratic Republic (Lao PDR) is a lower-middle-income country in Southeast Asia, bordering Thailand, Vietnam, Cambodia, China, and Myanmar. The population of the Lao PDR was 6.5 million in 2015, and 63 % of the population lives in rural areas [1]. The predominant crop is rice, and 71 % of the households in the nation were engaged in rice farming in 2010/11 [2]. Two-thirds of the land is either hilly or mountainous. The Lao PDR has a monsoonal climate. The rainy season extends from April to November, with peak rainfall in July and August [1, 2]. Although malaria cases are reported from healthcare facilities throughout the year, there is an increase in the number of reported cases during the rainy season [3]. *Plasmodium falciparum* has long been the predominant reported infection, but the incidence *Plasmodium vivax* has been increasing in recent years [4]. The groups most at risk of malaria infection in the Lao PDR include ethnic minorities, forest fringe inhabitants, migrant workers, and new forest settlers [3, 5, 6]. People in these groups live or work in the forest where they are engaged in cropping, hunting, collecting of forest products, mining, or constructing roads/dams. Therefore, their exposure to vector mosquitoes is increased. Among the ethnic minorities and forest fringe inhabitants, adult males in particular report that they stay in the forest overnight to hunt or collect wood [3, 7, 8].

Since 1992, the Lao PDR has implemented a nationwide malaria control programme [8]. The current malaria control strategies emphasize the promotion of long-lasting insecticide-treated bed nets, early diagnosis by microscopic examination and rapid diagnostic tests (RDT), and prompt treatment with an artemisinin-based combination therapy (ACT). Since 2008, the use of ACT has gradually been scaled-up to cover the whole public health sector, including village health volunteers (community health workers) and some businesses in the private sector, including registered private pharmacies [3].

As a result of the above-mentioned control efforts, there have been remarkable decreases in the rates of mortality and morbidity due to malaria over the past decade. The Lao PDR achieved a >75 % decrease in the incidence of malaria between 2000 and 2015. However, asymptomatic infections continue to be detected in rural, remote villages [7, 9, 10], which contributes to on-going local transmission. Asymptomatic carriers are not detected by the current national surveillance system, which is based on passive case detection at healthcare facilities. Furthermore, patients who are symptomatic may not necessarily seek treatment from healthcare facilities in which the surveillance system is in place [11]. Thus, the actual intensity of malaria transmission in the communities of the Lao PDR remains poorly understood.

Artemisinin resistance has been increasingly reported from many areas in the Greater Mekong Sub-region (Lao PDR, Cambodia, Myanmar, Thailand, Vietnam, and China) [12], posing a threat to regional and global health security. In 2014, the Malaria Policy Advisory Committee to the World Health Organization reviewed the situation of the region, considered malaria elimination as technically and operationally feasible, and thus recommended that the regional countries adopt a goal of malaria elimination by 2030 [13]. The elimination strategies in each country should be based on the local epidemiology [13]. However, information is lacking to guide and implement elimination strategies in the Lao PDR. A community-based survey with a highly sensitive molecular measurement method [i.e., polymerase chain reaction (PCR)] can help to better understand the epidemiological trends in the communities of the Lao PDR.

Approximately 90 % of all malaria cases in the Lao PDR occur in the southernmost provinces including Savannakhet province [3]. Of the 15 districts in this province, malaria is endemic in four districts including Xepon district. Xepon district is a remote, rural district on the Vietnamese border, approximately 500 km from the national capital of Vientiane. Ethnic minorities comprise 75 % of the total district population, which is approximately 45,000. There are 88 villages in the district, most of which are at risk of malaria. According to the Xepon District Health Office, 163 malaria cases were reported from the Xepon District Hospital in 2013, including 151 cases of *P. falciparum* mono-infection, 10 cases of *P. vivax* mono-infection, and two cases of co-infections. In 2012, 218 cases were reported, including 214 cases of *P. falciparum* mono-infection and four cases of *P. vivax* mono-infection. Xepon district was deliberately selected because reliable demographic data were available on households in the 20 villages in which the Health and Demographic Surveillance System (HDSS) was in place. The HDSS has been described elsewhere [14].

The primary objective of this study was to explore the characteristics of individuals with malaria infections in a rural district of the Lao PDR. The specific objectives were (1) to investigate the prevalence and species of malaria parasites in the study community using molecular methods, and (2) to assess the individual- and household-level characteristics that are associated with malaria infection.

## Methods

### Study site and population

**Fig. 1:**
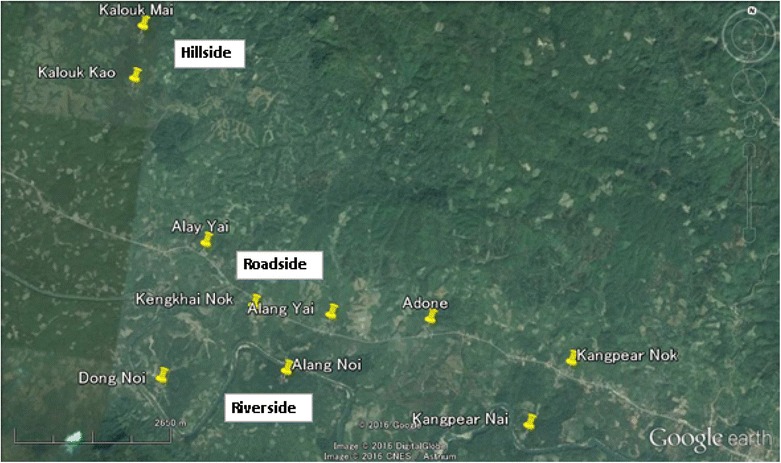
Map of the study villages. *Yellow pins* indicate the locations of the study villages

This study was conducted in Xepon district, Savannakhet province, Lao PDR between August and September of 2013. From the 20 villages where the HDSS was in place, 10 villages were deliberately selected as the study site based on the geographical characteristics of these villages: two hillside villages, five roadside villages, and three riverside villages were selected (Fig. 1). Data were collected from 870 participants in 236 households (participation rate: 47.8 %).

### Data collection

Data were collected through a cross-sectional, community-based survey. The survey consisted of interviews, blood examinations, and body temperature measurements. All of the villagers in each study village were invited to assemble at a designated place, including a primary school and village health volunteers’ houses. First, trained surveyors, who were nurses or medical technologists, identified a participant by his/her name, the name of his/her household head, and the household ID used for the HDSS. Second, surveyors conducted an interview using a pre-tested questionnaire. In the case of child participants, a guardian responded to the interview. Third, the surveyors measured the body temperature of the participants with a digital ear thermometer (MC-510, Omron Healthcare Co., Ltd., Kyoto, Japan). Finally, the surveyors collected a finger-prick blood sample from the participants that was subjected to RDT (SD Bioline Malaria Ag Pf/Pv, Standard Diagnostics, Inc., Gyeonggi-do, Republic of Korea). Blood samples were also collected for microscopic examination and PCR. For PCR, blood samples were collected on Whatman FTA Classic Cards (GE Healthcare Life Science, Little Chalfont, UK).

### Laboratory procedures

#### Microscopy

Experienced medical technologists prepared Giemsa-stained thick and thin blood smears and performed the microscopic examinations.

#### DNA extraction and PCR

Deoxyribonucleic acid (DNA) was extracted from dried blood spots on the FTA Classic Cards with a QIAamp DNA Mini Kit (Qiagen, Hilden, Germany) in accordance with the manufacturer’s instructions. The extracted DNA was eluted with 50 µl of elution buffer in the kit and preserved at 4 °C until the PCR. The PCR was performed in two steps. First, screening by a real-time PCR was performed with genus-specific primers targeting the *cytochrome b* gene in the mitochondrial genome of *Plasmodium* parasites [15]. Second, *Plasmodium* species identification was performed with species-specific primer sets targeting the same gene by a nested PCR with DNA samples that were identified as being *Plasmodium* parasite-positive in the screening. SaoAdvanced Universal SYBR Green Supermix (Bio-Rad, CA, USA) was used for the screening PCR with 2 µl of the extracted DNA and the primer set (Additional file 1). When the melting temperature of the PCR product was between 76.5 and 78.5 °C, the sample was considered to be *Plasmodium* DNA positive.

For the PCR for species identification, primer sets for *P. falciparum*, *P. vivax*, *Plasmodium malariae*, and *Plasmodium ovale* were used. The primary PCR for the identification was conducted by the real-time PCR. The secondary PCR for the identification was conducted using a conventional PCR with the first PCR product diluted 1000 times with Go-*Taq* DNA Polymerase (Promega, Fitchburg, WI, USA). The PCR products were visualized by 2 % agarose gel electrophoresis with Gel Red (Biotium, Fremont, CA, USA) staining by UV transillumination. All of the DNA extraction procedures and PCRs were conducted at the National Center for Global Health and Medicine, Japan.

#### Factors and measurements

The primary outcome measure in this study was the malaria infection status principally determined by PCR. The secondary outcome measure was the malaria infection status determined by RDT and then confirmed principally by PCR, i.e., true positives detected by RDT. Malaria infection was defined by a positive test result with or without clinical symptoms. The individual-level predictive factors included age, gender, bed net use on the night before the survey, the experience of working in the forest at night during the previous month, and the experience of a fever during the two weeks prior to the survey. The age and gender variables were determined from the information in the HDSS database because the self-reported ages of the subjects at the study site might not have been reliable. Other variables were measured using a questionnaire-based interview. At the household level, the predictive factors included the number of household members, the person-per-net ratio (defined as the number of household members divided by the number of available bed nets in a household), and living in a household with one other malaria-infected member. These household-level factors were determined according to the information in the HDSS database and the results of a bed net observation survey that was conducted prior to the present study [16].

### Statistical analysis

For the analysis of associations between the outcomes and predictive factors, multi-level modelling was used to account for the hierarchical structure of the data; i.e., 870 individuals in 236 households. Thus, a two-level, mixed-effects logistic regression model was used for the multivariate analysis. For the bi-variate analysis, a Chi square test or Fisher’s exact test was used. For other statistical analyses, a Chi square test or Fisher’s exact test was used for a categorical variable and a t test was used for a discrete variable. A *p* < 0.05 was considered statistically significant. All statistical analyses were performed with the STATA MP12 software program (StataCorp LP, College Station, TX, USA).

## Results

### Infection characteristics

Fifty-two participants were confirmed to have malaria parasitaemia: 49 individuals were confirmed by PCR and the other three individuals, whose filter paper samples were not available, were determined by microscopic examination (Table 1). The total positive rate was 6.0 % (52/870). Mono-infection with *P. falciparum* was the most common form of infection (n = 41, 78.8 %), followed by mixed infection with *P. falciparum* and *P. vivax* (n = 9, 17.3 %). RDT and microscopic examinations could only detect 36.7 % of the PCR-diagnosed infections.

**Table 1:** Blood examination results

	n	%
*Species*		
Total (n = 52)		
*P. falciparum* mono-infection	41	78.8
*P. vivax* mono-infection	2	3.8
*P. falciparum* and *P. vivax* mixed infection	9	17.3
PCR positives (n = 49)		
*P. falciparum* mono-infection	38	77.6
*P. vivax* mono-infection	2	4.1
*P. falciparum* and *P. vivax* mixed infection	9	18.4
Rapid diagnostic test positives (n = 21)		
*P. falciparum* mono-infection	19	90.5
*P. vivax* mono-infection	1	4.8
*P. falciparum* and *P. vivax* mixed infection	1	4.8
Microscopic examination positives (n = 21)		
*P. falciparum* mono-infection	18	85.7
*P. vivax* mono-infection	3	14.3
*P. falciparum* and *P. vivax* mixed infection	0	0.0
Sensitivity of rapid diagnostic test against PCR positives		
Positive	18	36.7
Negative	31	63.3
Sensitivity of microscopic examination against PCR positives		
Positive	18	36.7
Negative	31	63.3

### Characteristics of individuals

Approximately one half of the participants (54.0 %) were female (Table 2), and nearly one half of the participants (47.2 %) were over 15 years of age. Bed net use was very common, and almost all of the participants (99.2 %) reported that they slept under a bed net on the night before the survey. Most of the participants did not work in the forests at night (96.2 %) and had not slept in forest/hut (99.1 %) over the past month. Most of the participants (91.8 %) had not experienced a fever episode in the past 2 weeks; this was also the case for the participants who showed positive test results (92.3 %). Most of the participants (77.2 %) did not have a measurable fever; this was also the case among the participants who showed positive test results (82.7 %).

**Table 2:** Characteristics of the participants

Characteristics	Total (n = 870)		Positive (n = 52)		Negative (n = 818)	
	n	%	n	%	n	%
Gender						
Female	470	54.0	24	47.1	446	54.5
Male	400	46.0	27	52.9	373	45.5
Age (years)						
<5	67	7.7	1	1.9	66	8.1
5–14	392	45.1	25	48.1	367	44.9
≥15	411	47.2	26	50.0	385	47.1
Slept under a bed net last night						
Yes	863	99.2	52	100.0	811	99.1
No	7	0.8	0	0.0	7	0.9
Worked in forest at night in the past month						
Yes	33	3.8	2	3.8	31	3.8
No	837	96.2	50	96.2	787	96.2
Slept in forest/hut in the past month						
Yes	8	0.9	1	1.9	7	0.9
No	862	99.1	51	98.1	811	99.1
Fever episode in the past 2 weeks						
Yes	71	8.2	4	7.7	67	8.2
No	799	91.8	48	92.3	751	91.8
Body temperature (°C)						
≤37	682	77.2	43	82.7	629	76.9
>37	198	22.8	9	17.3	189	23.1
Mean (standard deviation)	36.8 (0.4)		36.8 (0.6)		36.8 (0.4)	

The majority (n = 42, 80.8 %) of the participants with positive test results did not have a fever episode in the two weeks before the survey or a measurable fever (>37 °C) at the time of the survey (Additional file 2).

### Demographic differences between participants and non-participants

There were significant differences in gender and age between the study participants and non-participants in the study villages (Table 3). The proportion of males was significantly lower in the participants than in the non-participants (46.0 vs 51.8 %, *p* = 0.012). The proportion of the population aged over 14 years of age was significantly lower in the participants than in the non-participants (47.1 vs 60.1 %, *p* < 0.001). There was also a difference in gender proportion between the participants and non-participants in the age subgroups: among the participants, males accounted for 53.7 % (36/67) in the youngest group, 49.7 % (195/392) in the second youngest group, and 40.6 % (167/411) in the oldest group. In contrast, among the non-participants, males accounted for 51.5 % (102/198) in the youngest group, 45.3 % (82/181) in the second youngest group, and 54.0 % (309/572) in the oldest group.

**Table 3:** Differences in demographic characteristics between participants and non-participants

Demographic characteristics	Total (n = 1821)		Participants (n = 870)		Non-participants (n = 951)		*P* value^a^
	n	%	n	%	n	%	
Gender							
Female	928	51.0	470	54.0	458	48.2	0.012
Male	893	49.0	400	46.0	493	51.8	
Age (years)							
<5	265	14.6	67	7.7	198	20.8	<0.001
5–14	573	31.5	392	45.1	181	19.0	
≥15	983	54.0	411	47.1	572	60.1	

^a^Chi square test

### Household characteristics

Most households (89.4 %) possessed at least one bed net (Table 4). According to the person-per-net ratio, 53.0 % of the households were considered to have a sufficient number of bed nets to cover all members of the household. Among the households with at least one infected person, 57.5 % of the households were considered to have a sufficient number of bed nets. The mean number of household members was 6.5.

**Table 4:** Characteristics of the households

Characteristics	Total (n = 236)		Households with at least one positive participant (n = 40)		Households without a positive participant (n = 196)	
	n	%	n	%	n	%
Possession of bed net						
Yes	211	89.4	35	89.7	176	89.3
No	4	1.7	0	0.0	4	2.0
Unknown	21	8.9	4	10.3	17	8.6
Person-per-net ratio						
≤2.5	125	53.0	23	57.5	102	52.0
>2.5 and <4.0	50	21.2	9	22.5	41	20.9
≥4.0 or no net	39	16.5	4	10.0	35	17.9
Unknown	22	9.3	4	10.0	18	9.2
Number of members						
<5	57	24.2	8	20.0	49	25.0
5–7	104	44.1	17	42.5	87	44.4
≥8	75	31.8	15	37.5	60	30.6
Mean (standard deviation)	6.5 (2.8)		6.8 (2.5)		6.4 (2.9)	

Among the 40 households with at least one infected member, nine households with at least two infected members were responsible for 40.4 % of the total infections (Additional file 3). Among these nine households, four households (44.4 %) had a sufficient number of bed nets. In contrast, among the 27 households that had only one infected member and that provided bed net data, 19 households (70.4 %) had a sufficient number of bed nets. Although there was a tendency for the households with two or more infected members to be less likely to have a sufficient number of bed nets as compared to the households with only one infected member, the difference (44.4 vs 70.4 %) was not statistically significant (*p* = 0.235 by Fisher’s exact test). Among the nine households that had at least two infected members, the mean number of members (standard deviation) was 7.2 (2.6), whereas it was 6.7 (2.5) among the 31 households that had only one infected member. The difference (7.2 vs 6.7) was not statistically significant (*p* = 0.574 by t test).

### Village characteristics

With the exception of one village, all of the villages had at least one malaria-positive participant (Table 5). The malaria positive rate, which ranged from 0 to 20.8 %, differed markedly among the villages (median: 6.0 %). The highest positive rate was found in the riverside villages. However, infected individuals were also found in hillside and roadside villages.

**Table 5:** Characteristics of the villages

Village name	Number of participants	Number of participants who tested positive	Positive rate (%)
Hillside			
Kalouk Mai	46	1	2.2
Kalouk Kao	115	12	10.4
Roadside			
Kangkai Nok	98	6	6.1
Adone	121	7	5.8
Alang Yai	93	7	7.5
Alay Yai	66	0	0.0
Kangpear Nok	104	1	1.0
Riverside			
Kangpear Nai	70	6	8.6
Alang Noi	53	11	20.8
Dong Noi	104	1	1.0

### Factors associated with malaria infection

#### Association with malaria infection status principally determined by PCR

The bivariate analysis showed that living in a household with at least one additional infected member was significantly associated with malaria infection (*p* < 0.001) (Table 6). No other variables were found to be associated with malaria infection. Consistent with the results of the bivariate analysis, the multivariate analysis showed that living in a household with at least one additional infected member was significantly associated with an increased risk of infection (adjusted odds ratio 24.33, 95 % confidence interval 10.15–58.32) (Table 7).

**Table 6:** Bivariate analysis of associations between malaria infection status principally determined by PCR and predictive factors

Predictive factors	Positive rate (%)	*P* value^a^
Individual level factors		
Age (years)		
<5	1.5	0.273
5–14	6.4	
≥15	6.3	
Gender		
Female	5.1	0.240
Male	7.0	
Working forest at night		
No	6.0	0.984
Yes	6.1	
Fever episode in the last 2 weeks		
No	6.0	1.000
Yes	5.6	
Household level factors		
Person-per-net ratio		
≤2.5	6.2	0.243
>2.5 and <4.0	7.2	
≥4.0 or no net	3.2	
Unknown	6.7	
Number of household members		
<5	7.6	0.689
5–7	5.4	
≥8	6.0	
Household with at least one additional infected member		
No	3.8	<0.001
Yes	45.6	

^a^Chi square test or Fisher’s exact test

**Table 7:** Multivariate analysis of associations between malaria infection status principally determined by PCR and predictive factors

Predictor variables	Adjusted odds ratio^a^	Adjusted 95 % confidence interval	*P* value
Individual level variables			
Age (years)			
<5	1.00	Reference	
5–14	5.17	0.59–45.10	0.137
≥15	4.42	0.51–38.52	0.179
Gender			
Female	1.00	Reference	
Male	1.14	0.60–2.17	0.689
Working forest at midnight			
No	1.00	Reference	
Yes	1.62	0.33–8.09	0.555
Fever episode in the last 2 weeks			
No	1.00	Reference	
Yes	1.32	0.39–4.46	0.656
Household level variables			
Person-per-net ratio			
≤2.5	1.00	Reference	
>2.5 and <4.0	0.62	0.26–1.49	0.287
≥4.0 or no net	0.59	0.19–1.83	0.360
Number of household members			
<5	1.00	Reference	
5–7	0.67	0.27–1.68	0.390
≥8	0.68	0.24–1.92	0.469
Household with at least one additional infected member			
No	1.00	Reference	
Yes	24.33	10.15–58.32	<0.001

^a^Adjusted for age, gender, working in forest, fever episode, person per net ratio, number of household members, and at least one additional infected member

#### Association with malaria infection status determined by rapid diagnostic tests

The bivariate analysis showed that living in a household with at least one additional infected member was significantly associated with malaria infection (*p* < 0.001 by Fisher’s exact test) (Table 8). No other variables were found to be significantly associated with malaria infection. However, there was a tendency for the positive participants to be more likely to have reported a fever episode as compared to the negative participants (19.0 vs 7.9 %, *p* = 0.084 by Fisher’s exact test). Consistent with the results of the bivariate analysis, the multivariate analysis showed that living in a household with at least one additional infected member was significantly associated with an increased risk of infection (adjusted odds ratio 56.69, 95 % confidence interval 9.38–342.61) (Table 9).

**Table 8:** Bivariate analysis of associations between malaria infection status determined by rapid diagnostic tests and predictive factors

Predictive factors	Positive (n = 21)		Negative (n = 849)		Positive rate (%)	*P* value^a^
	n	%	n	%		
Individual level factors						
Age (years)						
<15	14	66.7	445	52.4	3.1	0.196
≥15	7	33.3	404	47.6	1.7	
Gender						
Female	11	52.4	459	54.1	2.3	0.879
Male	10	47.6	390	45.9	2.5	
Working forest at night						
No	20	95.2	817	96.2	2.4	0.560
Yes	1	4.8	32	3.8	3.0	
Fever episode in the last 2 weeks						
No	17	81.0	782	92.1	2.1	0.084
Yes	4	19.0	67	7.9	5.6	
Household level factors						
Person-per-net ratio						
≤2.5	8	38.1	412	48.3	1.9	0.230
>2.5 and <4.0	9	42.9	227	26.7	3.8	
≥4.0 or no net	2	9.5	154	18.1	1.3	
Unknown	2	9.5	58	6.8	3.3	
Number of household members						
<5	0	0.0	119	14.0	0.0	0.171
5–7	11	52.4	376	44.3	2.8	
≥8	10	47.6	354	41.7	2.7	
Household with at least one additional infected member						
No	16	76.2	839	98.8	1.9	<0.001
Yes	5	23.8	10	1.2	33.3	

^a^Chi square test or Fisher’s exact test

**Table 9:** Multivariate analysis of associations between malaria infection status determined by rapid diagnostic tests and predictive factors

Predictor variables	Adjusted odds ratio^a^	Adjusted 95 % confidence interval	*P* value
Individual level variables			
Age (years)			
<15	1.00	Reference	
≥15	0.52	0.18–1.46	0.211
Gender			
Female	1.00	Reference	
Male	1.01	0.38–2.69	0.990
Working forest at midnight			
No	1.00	Reference	
Yes	2.27	0.26–20.09	0.461
Fever episode in the last 2 weeks			
No	1.00	Reference	
Yes	2.54	0.73–8.87	0.143
Household level variables			
Person-per-net ratio			
≤2.5	1.00	Reference	
>2.5 and <4.0	1.01	0.29–3.52	0.986
≥4.0 or no net	0.62	0.13–3.02	0.558
Number of household members			
<8	1.00	Reference	
≥8	0.38	0.10–1.42	0.152
Household with at least one additional infected member			
No	1.00	Reference	
Yes	56.69	9.38–342.61	<0.001

^a^Adjusted for age, gender, working in forest, fever episode, person-per-net ratio, number of household members, and at least one additional infected member

## Discussion

As expected from the patient records of the Xepon District Hospital, the present community-based study showed that *P. falciparum* mono-infection accounted for the majority of infections (78.8 %). However, the results of the present study also showed a difference in the proportion of *P. falciparum* and *P. vivax* infections in the patient records and the present study: *P. vivax* infections including co-infections with *P. falciparum*, which were rarely reported from the hospital in 2012 (1.8 %) and 2013 (7.9 %), accounted for 21.2 % of the infections that were detected in the present study. This suggests that the burden of vivax malaria is underestimated in the district.

Consistent with the findings of a previous study conducted in Savannakhet province [9], the present study found that most of the infections (63.3 %) were asymptomatic and sub-microscopic. This finding highlighted the importance of tackling asymptomatic infections or “hidden malaria” in malaria elimination efforts in the malaria-prone districts of the Lao PDR. Thus far, no specific strategy for identifying asymptomatic parasite carriers has been implemented in the Lao PDR. Therefore, the introduction of active case detection for asymptomatic infections is recommended.

The results of the present study indicated that infections were highly clustered at the household level and that infections were unevenly distributed across households. A considerable number of studies have reported household clustering in countries other than the Lao PDR [17–19]. One possible explanation for the household clustering is that household members share the same environmental risk factors including the proximity of housing location to breeding sites [20] and housing type [21]. Another possible explanation is that household members share the same risk behaviors such as not using a bed net [9, 20] and working in the forest [10]. The findings of the present study suggest the implementation of household-based strategies to achieve effective active case detection. A reactive case detection strategy that is linked to facility-based passive case detection can be an option for actively detecting cases in a setting where infections are highly clustered within households and the transmission intensity is low [22]. As reported elsewhere [23, 24], malaria patients who are identified through facility-based passive case detection can be used as an index case. Subsequently, individuals living with the index case are screened.

For such reactive case detection to be implemented in Xepon district or other similar settings in the Lao PDR, RDT would be used as a screening tool. Although the present study showed that RDT detected only 36.7 % of the true positives confirmed by PCR in the study site and malaria elimination cannot be achieved by screening with the current generation of RDTs [25], the use of PCR for screening is not currently feasible in the rural districts of the Lao PDR. To increase the detection rate of secondary infections by screening with RDT, the following strategies should be incorporated. First, screening should focus on the households of index cases that do not possess a sufficient number of bed nets. In the present study, the households with multiple cases of infection were less likely to have a sufficient number of bed nets than were the households with a single case, although the difference was not statistically significant, possibly because of the small sample size (n = 36). Second, screening should focus on the households of index cases in which a household member has a history of fever/malaria. The present study found that two-week recall of a fever episode was reported only from the participants who had a RDT detectable infection. The present study also found that the RDT-positive participants were more likely to have reported a fever episode than were the RDT-negative participants (19.0 vs 7.9 %), although the difference was not significant. A previous study conducted in Savannakhet province reported that malaria-related symptoms in the past one year and a history of clinical malaria were predictive factors for the PCR-diagnosed malaria infections [9]. A Cambodian study [23] that assessed a reactive case detection approach using index cases found through passive case detection at a health facility or through a village malaria worker reported a significant association between the secondary infections detected by RDT and measured fever (>37.2 °C) and previous malaria. Finally, screening should focus on the households of index cases with an environmental risk factor. Another study conducted in the Lao PDR showed that the number of Anopheles mosquitoes that entered people’s homes differed significantly according to the type of house, the presence of cattle, and the presence of wood smoke from cooking fires [21].

The Cambodian study [23] showed, however, that the reactive case detection approach identified very few secondary infections. Among the members of the index cases, the positive rate of the secondary infections was 0.3 % by RDT and 1.3 % by PCR. The study recommended that the reactive case detection approach is not appropriate for very low transmission settings in which exposure to malaria occurs away from the community and there is a high level of treatment-seeking from the private sector. Given this recommendation, the reactive case detection approach could be appropriate in the study district of Xepon because one half of the infections occurred in children, who accounted for 52.8 % of the participants, and a substantial proportion of the infections might have occurred within the village or on the fringe of the village. The possibility of such local transmission around the village can be supported by the findings of another study conducted in three villages in Xepon and Nong districts, Savannakhet province [10]. The study showed that many *Anopheles dirus* mosquitoes, which are a main vector in the Lao PDR, were captured in indoor light traps. Although treatment seeking from the private sector is also high in Xepon district, in which approximately 44 % of the malaria cases were reported from the private sector in 2013/14, the private sector has been an integral part of the National Malaria Control Programme in the Lao PDR including Xepon district. Most importantly, there was a marked difference in the transmission intensity between the present study and the Cambodian study (positive rate by RDT: 2.4 vs 0.5 %).

A number of factors that were previously reported as risk factors from the Lao PDR and/or neighboring countries were not identified as risk factors in the present study site. Although not using a bed net was reported as a risk factor for malaria infection from Savannakhet province [9], almost all of the participants (99.2 %) in the present study reported use of a bed net on the night before they were surveyed. Likewise, although sleeping away from home was reported as a risk factor for malaria infection from Attapeu province, Lao PDR [20], very few of the present participants (0.9 %) did this. Additionally, working in the forest at night, which is a known risk factor in Vietnam and Thailand [26, 27], was not common in the present study site. Because the present study failed to include more than one half of the population in the study site, it could possibly identify these previously reported risk factors if most of the population were included. For example, because the present study found that only 53.0 % of the households owned a sufficient number of bed nets to fully cover all members, some of the non-participants might not have used a bed net.

A major limitation of this study is the low participation rate: only 47.8 % of the population in the study villages participated. As Table 3 shows, compared to the participants, the non-participants were more likely to be male, children under the age of five, and adults (especially adult males). A possible reason for the lower participation rate among the children is that their guardians are unwilling to expose their young children to blood collection. A possible reason for the lower participation rate of the adults is that they worked outside the village during the period of data collection. Because of this limitation, the results of the present study could underestimate the risk of malaria infection among young children and adults while overestimating the risk among older children.

This study has two other limitations. First, because the data collection was confined to half of the population in the 10 villages from the HDSS area, the present sample was not representative of the population of Xepon district. Second, the survey took place between August and September (the rainy season) and, therefore, did not take seasonal malaria epidemiology into account or seasonal variation in the frequency of risk factors such as sleeping away from home, working in the forest at night, and not sleeping under a bed net. However, sleeping away from home is more frequently reported in the rainy season than in the dry season in the Lao PDR [15].

## Conclusions

The present community-based study showed that *P. falciparum* infections were common but that infections involving *P. vivax*, which was rarely reported from the district healthcare facility, accounted for 21 % of the malaria infections. This suggested that the burden of *P. vivax* infection is underestimated. Most (63 %) of the infections were asymptomatic and sub-microscopic and were highly clustered within households. To eliminate malaria in Xepon district and other similar settings in the Lao PDR, the National Malaria Control Programme should consider household-based control strategies. The findings of the present study suggest the use of a reactive case detection strategy targeting the household members of index cases.

## Additional files

**Additional file 1.** Primer sequences.

**Additional file 2.** Characteristics of each participant with positive test results.

**Additional file 3.** Characteristics of each household with a positive participant.

## Abbreviations

Lao PDR: The Lao People’s Democratic Republic
RDT: rapid diagnostic test
ACT: artemisinin-based combination therapy
PCR: polymerase chain reaction
HDSS: health and demographic surveillance system
DNA: deoxyribonucleic acid
