# Supplementary material for: Household clustering of asymptomatic malaria infections in Xepon district, Savannakhet province, Lao PDR
Source: Malar J. 2016 Oct 18;15:508. doi: 10.1186/s12936-016-1552-7 (PMC5069939; doi:10.1186/s12936-016-1552-7)
Supplement: Supplementary file 1 — Additional file 1. Primer sequences. [file 12936_2016_1552_MOESM1_ESM.docx]

**Additional file 1 Primer sequences**

| **Type of PCR** | **Primer name** | **Primer sequences (5' --> 3')** | **Species** | **Target gene** | **Reference** |
| --- | --- | --- | --- | --- | --- |
| Real-time PCR for screening |  |  |  |  |  |
|  | RTPCRScreening2_F | TGGAGTGGATGGTGTTTTAGA | *Plasmodium spp.* | *cytochrome b* | 15 |
|  | RTPCRScreening2_R | TTGCACCCCAATARCTCATTT | *Plasmodium spp.* | *cytochrome b* | 15 |
| Nested PCR for identification |  |  |  |  |  |
| Primary PCR (Real-time) | Sc2_F | TGGAGTGGATGGTGTTTTAGA | *Plasmodium spp.* | *cytochrome b* | 15 |
| Primary PCR (Real-time) | Sc3_R | ACCCTAAAGGATTTGTGCTACC | *Plasmodium spp.* | *cytochrome b* | 15 |
| Secondary PCR (Conventional) | Pf_nest_R12 | TCATTTGACCCCATGGTAAGA | *P. falciparum* | *cytochrome b* | Designed in this assay |
| Secondary PCR (Conventional) | Pf_nest_F11 | AGATACATGCACGCAACAGG | *P. falciparum* | *cytochrome b* | Designed in this assay |
| Secondary PCR (Conventional) | PvR | ATTTGTCCCCAAGGTAAAACG | *P. vivax* | *cytochrome b* | 15 |
| Secondary PCR (Conventional) | PvF | TGCTACAGGTGCATCTTGTATTC | *P. vivax* | *cytochrome b* | 15 |
| Secondary PCR (Conventional) | PmF | ACAGGTGCATCACTTGTATTTTTTC | *P. malariae* | *cytochrome b* | 15 |
| Secondary PCR (Conventional) | PmR | TGCTGGAATTGAAGATAATAAATTAGTAATAACT | *P. malariae* | *cytochrome b* | 15 |
| Secondary PCR (Conventional) | PoF | GTTATATGGTTATGTGGAGGATATACTGTT | *P. ovale* | *cytochrome b* | 15 |
| Secondary PCR (Conventional) | PoR | CGAATGGAAGAATAAAATGTAGTACG | *P. ovale* | *cytochrome b* | 15 |

Real-time: real-time PCR, Conventional: conventional PCR
