# Supplementary material for: Household clustering of asymptomatic malaria infections in Xepon district, Savannakhet province, Lao PDR
Source: Malar J. 2016 Oct 18;15:508. doi: 10.1186/s12936-016-1552-7 (PMC5069939; doi:10.1186/s12936-016-1552-7)
Supplement: Supplementary file 2 — Additional file 2. Characteristics of each participant with positive test results. [file 12936_2016_1552_MOESM2_ESM.docx]

**Additional file 2 Characteristics of each participant with positive test results**

| Participant ID | Gender | Age | Fever episode in the past two weeks | Body temperature | Species* |
| --- | --- | --- | --- | --- | --- |
| 1 | Female | 24 | No | 36.5 | Pf |
| 2 | Male | 9 | No | 37.0 | Pf |
| 3 | Male | 9 | Yes | 37.9 | Pf |
| 4 | Male | 7 | Yes | 38.2 | Mix |
| 5 | Male | 5 | No | 36.9 | Pf |
| 6 | Female | 27 | No | 37.6 | Mix |
| 7 | Female | 11 | No | 37.9 | Pf |
| 8 | Female | 13 | No | 36.9 | Pf |
| 9 | Male | 7 | No | 37.0 | Pf |
| 10 | Female | 40 | Yes | 37.0 | Pf |
| 11 | Male | 40 | No | 36.6 | Pf |
| 12 | Female | 7 | No | 36.7 | Pf |
| 13 | Male | 39 | No | 36.7 | Pf |
| 14 | Female | 10 | No | 36.3 | Pf |
| 15 | Female | 4 | No | 37.0 | Pf |
| 16 | Male | 63 | No | 37.0 | Mix |
| 17 | Male | 60 | No | 37.0 | Pf |
| 18 | Female | 5 | No | 36.7 | Mix |
| 19 | Female | 30 | No | 36.9 | Mix |
| 20 | Male | 19 | No | 36.7 | Pf |
| 21 | Male | 43 | No | 36.9 | Mix |
| 22 | Male | 31 | No | 36.8 | Pf |
| 23 | Male | 7 | No | 36.0 | Pf |
| 24 | Female | 27 | No | 36.8 | Pf |
| 25 | Female | 8 | Yes | 37.2 | Mix |
| 26 | Male | 12 | No | 36.9 | Pf |
| 27 | Female | 10 | No | 36.8 | Pf |
| 28 | Female | 10 | No | 36.9 | Pv |
| 29 | Male | 63 | No | 36.4 | Pf |
| 30 | Male | 32 | No | 36.8 | Pf |
| 31 | Female | 30 | No | 36.0 | Pf |
| 32 | Male | 9 | No | 34.9 | Pf |
| 33 | Male | 8 | No | 37.3 | Pf |
| 34 | Female | 11 | No | 36.3 | Pf |
| 35 | Male | 6 | No | 36.7 | Pf |
| 36 | Male | 42 | No | 36.2 | Pf |
| 37 | Male | 57 | No | 36.6 | Pf |
| 38 | Male | 27 | No | 35.3 | Pf |
| 39 | Female | 27 | No | 36.9 | Pf |
| 40 | Female | 16 | No | 36.9 | Pf |
| 41 | Female | 14 | No | 36.9 | Pf |
| 42 | Male | 11 | No | 37.0 | Pf |
| 43 | Male | 7 | No | 37.0 | Pf |
| 44 | Male | 38 | No | 36.3 | Pv |
| 45 | Female | 10 | No | 36.5 | Pf |
| 46 | Male | 47 | No | 36.6 | Pf |
| 47 | Female | 37 | No | 36.5 | Pf |
| 48 | Female | 26 | No | 36.7 | Mix |
| 49 | Female | 7 | No | 37.0 | Pf |
| 50 | Male | 18 | No | 36.6 | Mix |
| 51 | Male | 30 | No | 36.9 | Pf |
| 52 | Female | 8 | No | 36.0 | Pf |

*****: Pf: *Plasmodium falciparum*, Pv: *Plasmodium vivax*, Mix: *P. falciparum* and *P. vivax* mixed infections
