# Supplementary material for: Household clustering of asymptomatic malaria infections in Xepon district, Savannakhet province, Lao PDR
Source: Malar J. 2016 Oct 18;15:508. doi: 10.1186/s12936-016-1552-7 (PMC5069939; doi:10.1186/s12936-016-1552-7)
Supplement: Supplementary file 3 — Additional file 3. Characteristics of each household with a positive participant. [file 12936_2016_1552_MOESM3_ESM.docx]

**Additional file 3 Characteristics of each household with a positive participant**

| Household ID | Number of members | Person per net ratio | Number of the positive participants | Species | Cumulative percent of the positive participants |
| --- | --- | --- | --- | --- | --- |
| 1 | 8 | 2.67 | 4 | Pf, Pf, Pf, Pf | 7.7 |
| 2 | 13 | 3.25 | 3 | Pf, Pf, Mix | 13.5 |
| 3 | 6 | 2.00 | 2 | Pf, Pf | 17.3 |
| 4 | 8 | 0.89 | 2 | Mix | 21.2 |
| 5 | 8 | 2.67 | 2 | Pf, Pf | 25.0 |
| 6 | 5 | 2.50 | 2 | Pf, Pf | 28.8 |
| 7 | 4 | 4.00 | 2 | Pf, Pf | 32.7 |
| 8 | 6 | 3.00 | 2 | Pf, Pf | 36.5 |
| 9 | 7 | 1.40 | 2 | Mix | 40.4 |
| 10 | 11 | 1.83 | 1 | Pf | 42.3 |
| 11 | 5 | 1.25 | 1 | Pf | 44.2 |
| 12 | 7 | 1.75 | 1 | Mix | 46.2 |
| 13 | 7 | 3.50 | 1 | Pf | 48.1 |
| 14 | 8 | 2.67 | 1 | Pf | 50.0 |
| 15 | 6 | 3.00 | 1 | Pf | 51.9 |
| 16 | 7 | 2.33 | 1 | Pf | 53.8 |
| 17 | 4 | 1.33 | 1 | Pf | 55.8 |
| 18 | 5 | 0.83 | 1 | Pf | 57.7 |
| 19 | 4 | 2.00 | 1 | Mix | 59.6 |
| 20 | 8 | 1.33 | 1 | Mix | 61.5 |
| 21 | 7 | 1.00 | 1 | Pf | 63.5 |
| 22 | 9 | 1.13 | 1 | Mix | 65.4 |
| 23 | 12 | 6.00 | 1 | Pf | 67.3 |
| 24 | 7 | 2.33 | 1 | Mix | 69.2 |
| 25 | 8 | 2.67 | 1 | Pf | 71.2 |
| 26 | 10 | 2.50 | 1 | Pv | 73.1 |
| 27 | 4 | 2.00 | 1 | Pf | 75.0 |
| 28 | 4 | 4.00 | 1 | Pf | 76.9 |
| 29 | 1 | 1.00 | 1 | Pf | 78.8 |
| 30 | 7 | 7.00 | 1 | Pf | 80.8 |
| 31 | 5 | 1.67 | 1 | Pf | 82.7 |
| 32 | 4 | 2.00 | 1 | Pf | 84.6 |
| 33 | 4 | 2.00 | 1 | Mix | 86.5 |
| 34 | 12 | 2.40 | 1 | Pf | 88.5 |
| 35 | 6 | 3.00 | 1 | Mix | 90.4 |
| 36 | 6 | 1.50 | 1 | Pf | 92.3 |
| 37 | 8 | Unknown | 1 | Pf | 94.2 |
| 38 | 8 | Unknown | 1 | Pf | 96.2 |
| 39 | 8 | Unknown | 1 | Pf | 98.1 |
| 40 | 5 | Unknown | 1 | Pf | 100.0 |

Pf: *Plasmodium falciparum*, Pv: *Plasmodium vivax*, Mix: *P. falciparum* and *P. vivax* mixed infections
